# Notes and Notices

**Published:** 1899-01

**Authors:** 


### NOTES AND NOTICES.

New York Medical College and Hospital.—Every graduate is requested to send his name and present address to the Corresponding Secretary of the Alumni Association in order that the new list of all graduates may be complete.

Dr. Edwin S. Munson,
*Corresponding Secretary.*
16 West 45th Street, New York.

Deadly Castor Oil Beans.—One child dies and three are made seriously ill.—The foreman of the coroner's jury, serving during this week's inquest, has made a record for himself on censures. To-day he incorporated one in the case of Ethel M. Kennedy, four years, 1336 Spangler Street, who died yesterday from the effect of eating castor oil beans, which she picked upon a lot near her home. They had been thrown there by a neighbor, who discarded them from her garden. The juryman wanted the owner of the lot censured for not having complied with the city ordinance, requiring all vacant plots to be enclosed. Deputy Coroner Dugan insisted that the matter was one in which the neighbors should first act by a petition to the Board of Health, as it came within the province of that department.

The father of the child said that other little ones in the neighborhood beside his own had partaken of the beans.

Dr. Cattell stated that from the symptoms of Ethel's illness, as detailed by the father, death had resulted from acute Bright's disease, induced by the beans. He had not made a post-mortem examination, as the mother was violently opposed to it. The active principle of the castor oil bean, explained the doctor, is ricine, a deadly poison, much used for commercial purposes.

The three Kennedy children partook of the beans on Tuesday afternoon. The other two, Thomas, aged five, and Albert, two years, are now suffering from Bright's disease, which ricine, if swallowed, produces in a violent form. Their condition was not as serious, however, as that of their sister, Ethel, and it is believed they will recover.—*Evening Bulletin*, Philadelphia, Nov. 17th, 1898.

The Mortality from Wounds and from Disease in the Cuban Campaign.—According to the *Philadelphia Medical Journal* for October 15th, while the mortality from wounds in the Cuban campaign as compared with that in the civil war was only as 2.3 per cent. to 15.3 per cent., indicating the great progress made in surgery in the interval, that from disease was 88.1 per cent. in the Cuban campaign as against 66.6 per cent. in the civil war, or an increase of nearly twenty-five per cent. While progress in medicine has not been so great, no doubt, as in surgery, still *there has been considerable progress*, and it would therefore only have been reasonable to look for some improvement in the mortality percentage. But here we are confronted by an increase instead of a decrease, and a considerable one at that. Now, in view of the fact that, as the *Philadelphia Medical Journal* points out, there has undoubtedly been some progress in medicine during the past thirty-three years, the reason for this must be sought in extra professional causes. What are these causes? This is a subject that demands the most earnest consideration of the investigating commission, and we trust it will get it. The *Philadelphia Medical Journal* has done a good work in calling attention to this fact.—*N. Y. Medical Journal*, October 22d, 1898.

Bœricke, Runyon & Ernesty.—The copartnership hitherto existing between Wm. Boericke, E. W. Runyon, and F. O. Ernesty has been dissolved by mutual consent; Mr. Ernesty retiring, having sold his interest and good-will to his partners, Dr. Wm. Boericke and Mr. E. W. Runyon, who will continue the business without any change or interruption at the present location, No. 497 Fifth Avenue. The new firm will be known as Bœricke & Runyon Co., and they assume all the assets and liabilities of the old firm.

See a lot of young girls in another column with their garments tucked up, treading grapes in a Quinto village of Portugal, during the wine-making season. The practice is kept up to this day. Speer, of New Jersey, however, uses rubber rollers, and makes the most superior wines of the world.

ALSO CLIMAX * * * BRANDY OF GRAPE

The superior vintage of 1878 brandy, introduced by the Speer N. J. Wine Co., is highly spoken of by physicians.

APPENDICITIS.—The following by Dr. H. A. Hare, of Philadelphia, clipped from a paper printed in *Dominion Medical Monthly* of September, must give pause to the cock-sure on appendicitis either way:

I have recently published in the *Medical News* some interesting cases of appendicitis, which show how one may be harassed by conflicting experiences. In one case I implored, besought, pleaded, and insisted that a young fellow with a history of nine attacks in six months should have an operation. He had an immense mass of inflammatory material about his appendix. He finally consented. One of the most eminent surgeons living operated. Stercoraceous vomiting speedily ensued, with collapse and death. I forced this man to an early death. In another instance I advised delay, because after this experience I had lost my nerve, for it came to my hands a few days after. Death met me again. Another case had a sharp attack of pain, with every classical sign of the disease. A surgeon said operate. The weather was excessively hot, the patient a feeble woman of fifty, and I felt sure the operation would kill her. I called a medical consultant who agreed with me. No operation was done, and the patient is now well and has had no attack since. I could go on with such cases indefinitely, and reach no clearer ideas as to the subject.

IN EUROPE SPEER'S PORT GRAPE WINE is ordered by families in Dresden, London, and Paris, for its superior medicinal virtues, and its blood-making quality. It is made from rare grape vines produced from Portugal.

ANTITOXIN.—The man who nightly puts his head in the yawning lion's mouth is reasonably sure that it will not be bitten off, yet he must always experience a slight trepidation. Similarly must those who inject antitoxin into children—if they read the medical journals. Dr. R. Abrahams (*Medical Record*) contributes his experience with this tricky and dangerous stuff as follows:

"F——, six years old, developed tonsillar diphtheria on a Friday. Saturday, at about five o'clock in the morning, I was called because croup appeared. The child was examined; diphtheritic membrane was found covering the tonsils. Although the child was decidedly croupy the breathing was far from alarming. Temperature, 102 degrees F., with a pulse in proportion. At half-past six o'clock I injected between the shoulder blades fifteen hundredth of a cubic centimetre of the antitoxine of the New York Board of Health. The reaction was marked by a rise of temperature of one degree, otherwise the child fell asleep, as most of them do after serum treatment. At eleven o'clock the temperature was the same, but there was evidence of improvement in the breathing and the cough. Nourishment was taken without protest. At one o'clock the report was still more gratifying. At two o'clock the child suddenly began to gasp for air, and became very blue, with cold extremities, poor pulse, and profuse cold perspiration. This alarming condition lasted but ten minutes. Then the child began to breathe quietly, pulse weak, and temperature sub-normal. A slow but gradual paralysis of the limbs and reflexes set in, and by five o'clock, in spite of the most vigorous treatment, the child died in a condition of total collapse and paralysis. The most curious feature of this case was that the quiet though slow breathing was preserved until the last moment, and, were it not for the ghastly appearance of the face, one could not, by mere inspection, tell the approach of death. There is no doubt in my mind, and in that of another physician whom I showed the case, that the antitoxin was responsible for the speedy and fatal termination."

Better stick to the homœopathic remedy, especially as it shows *far* better results than antitoxin at its best, and *never* kills the patient. —*Homœopathic Recorder for October.*

Potassium Permanganate in Morphine Poisoning.—*The Medical Visitor*, in its June number, makes a strong plea for the value of permanganate of potassa as an antidote in Morphine poisoning. Experiments on dogs show that one and a half grains of permanganate, hypodermically given, neutralized the toxic effects of two and one-half grains of Morphine.

---

### FUN FOR DOCTORS.

Vegetarian Doctrine.—"These vegetarians are all wrong."

"Think so?"

"Certainly, meat makes life."

"That's one doctrine perhaps."

"It's the truth. Didn't it take a spare rib to make Eve?"—*Indianapolis Journal.*

There came a young freshman to college.
When he heard that he had to get knowledge,
He said, "Goodness me!
Why, how can this be?
What a queer thing to do at a college!"

Sunday-school Teacher.—"Why, Willie Wilson! Fighting again! Didn't last Sunday's lesson teach that when you are struck on one cheek you ought to turn the other to the striker?"

Willie.—"Yes'm; but he hit me on the nose, an I've only got one."

